# Patterns and prognostic implications of cutaneous metastasis in Hong Kong: A multicenter analysis

**DOI:** 10.1016/j.jdin.2025.11.004

**Published:** 2025-11-14

**Authors:** Sau Yee Chan, Joanna Ka Man Ng, Agnes Wai Sze Chan, Christina Man-Tung Cheung, Paul Cheung Lung Choi, Ka Wun See, Joshua Jing Xi Li

**Affiliations:** a Department of Anatomical and Cellular Pathology, Prince of Wales Hospital, The Chinese University of Hong Kong, Hong Kong; b Department of Pathology, Alice Ho Miu Ling Nethersole Hospital, Hong Kong; c Department of Pathology, North District Hospital, Hong Kong; d Division of Medicine and Therapeutics, Prince of Wales Hospital, The Chinese University of Hong Kong, Hong Kong; e Department of Pathology, Queen Mary Hospital, The University of Hong Kong, Hong Kong

**Keywords:** cutaneous metastasis, epidemiology, metastatic breast cancer, Sister Mary Joseph nodule

*To the Editor:* Cutaneous metastasis is a rare but prognostically significant presentation and is associated with negative outcomes.^1^^,^^2^ We reviewed a multicenter cohort of cutaneous metastasis with histologic confirmation from a period of 27 years to identify prognostically predictive parameters and patterns of presentation and spread in cutaneous metastasis. Statistical analysis was performed using SPSS (version 26). The χ^2^ test was used to compare the sites for cutaneous metastasis and primary malignancy as categorical variables. Survival analysis for overall survival (OS) was performed using Kaplan-Meier analysis. Cox regression using the backward Wald method was used for multivariable analysis. A *P* < .05 was considered significant.

A total of 161 cases were retrieved. (78 males and 83 females, mean age 62 years). Follow-up data were available for 155 cases (mean follow-up of 23.6 months). The most common sites of cutaneous metastasis were chest (n = 34/161, 21.1%), scalp (n = 25/161, 15.5%), and abdomen (n = 21/161, 13.0%) (Fig 1). The most common sites of primary malignancy were the gastrointestinal tract (n = 46/161, 28.6%), breast (n = 34/161, 21.1%), and lung (n = 29/161, 18.0%). For females, the most common primary malignancy was breast (n = 33/83, 39.8%), and for males, gastrointestinal (n = 23/78, 29.5%) and lung (n = 23/78, 29.5%).

**Fig 1:**
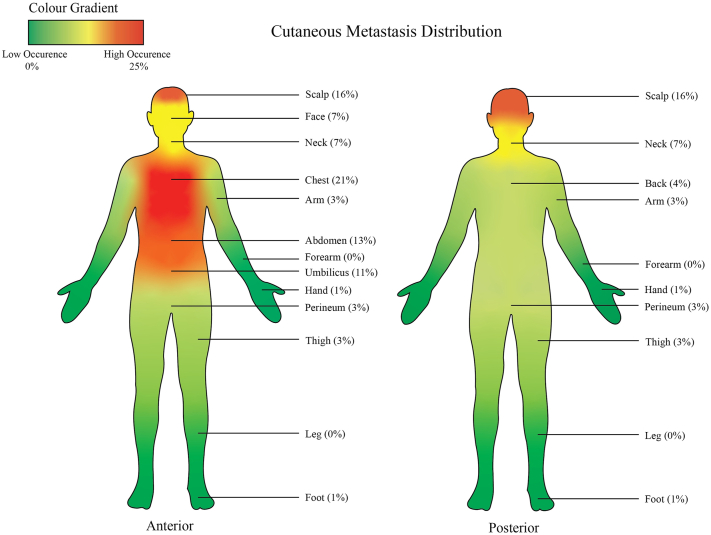
Distribution of cutaneous metastasis by site.

The most common pattern of spread was from abdominopelvic primaries to the torso (n = 40/161, 24.8%), then thoracic primaries to the torso (n = 39/161, 24.2%). Out of 17 cases of cutaneous metastasis to the umbilicus, most were from colorectal (7/17, 41.2%) and ovarian (n = 6/17, 35.3%) malignancies.

Kaplan-Meier analysis showed that male sex (*P* < .001) and age over 60 years old (*P* = .003) were associated with worse OS. Sites of primary malignancy in terms of organ (*P* < .001) and grouped analysis to superficial versus visceral sites (*P* = .004) both revealed statistically significant results, with breast having the longest OS of 74.4 months, female genital malignancies second at 33.0 months, and lung the shortest (8 months) (Table I). Superficial malignancies (breast, head and neck, and skin) demonstrated longer OS compared with visceral malignancies (*P* = .004. Among visceral malignancies, those from the abdomen and pelvis had longer OS (*P* = .005). Sites of cutaneous metastasis did not show statistically significant correlation with OS (*P* > .05). On multivariate analysis, male sex (*P* = .025) and age over 60 years old (*P* = .022) were independently associated with worse OS, while primary malignancy from the breast (*P* = .009) was independently associated with better OS.

**Table I:** Overall survival (Kaplan-Meier analysis)

	Survival (mo)	*P* value
Sex		
Male	15.5	<.001
Female	47.1	
Age		
<60 y	49.2	.003
≥60 y	20.6	
Site of cutaneous metastasis		
Head and neck	14.9	.093
Torso and extremities	37.1	.232
Torso	34.5	
Extremities	52.1	
Primary malignancy		
By organ site	74.4	<.001
Breast	33.0	
Female genital tract	17.0	
Gastrointestinal tract	20.0	
Genitourinary tract	10.7	
Head and neck	8.0	
Lung	36.8	
Carcinoma of unknown primary		
Superficial∗ vs visceral		
Superficial	45.4	.004
Visceral	23.7	.005
Thorax	9.3	
Abdomen and pelvis	26.0	

∗ Includes breast, head and neck, and cutaneous malignancies.

This study reviewed histology-proven cutaneous metastasis in multiple institutions over multiple decades, identifying prognostically significant clinicopathological parameters. The study is limited by the heterogeneity of treatment received throughout the recruitment period, with novel targeted therapies only available to patients presented in the recent decade. The patterns of metastasis and survival outcomes may be explained by the route (perineural or lymphovascular) of dissemination leading to cutaneous metastasis.^3^ Recognizing poor prognostic subgroups and stratifying favorable patients are important for expectation management and treatment planning.^4^ Medium-to-long survival is not infrequently seen in young female patients with primary female (breast and genital) malignancies. It would be beneficial to stratify prognostically distinct groups for early management planning.

## Conflicts of interest

None disclosed.
